# Composite P(3HB-3HV)-CS Spheres for Enhanced Antibiotic Efficiency

**DOI:** 10.3390/polym13060989

**Published:** 2021-03-23

**Authors:** Oana Gherasim, Alexandru Mihai Grumezescu, Anton Ficai, Valentina Grumezescu, Alina Maria Holban, Bianca Gălățeanu, Ariana Hudiță

**Affiliations:** 1 Department of Science and Engineering of Oxide Materials and Nanomaterials, Faculty of Applied Chemistry and Materials Science, Politehnica University of Bucharest, 011061 Bucharest, Romania; oana.fufa@gmail.com (O.G.); grumezescu@yahoo.com (A.M.G.); anton.ficai@upb.ro (A.F.); 2 Lasers Department, National Institute for Lasers, Plasma, and Radiation Physics, RO-77125 Magurele, Romania; 3 Research Institute of the University of Bucharest—ICUB, University of Bucharest, 050657 Bucharest, Romania; 4 Microbiology & Immunology Department, Faculty of Biology, University of Bucharest, 77206 Bucharest, Romania; alina_m_h@yahoo.com; 5 Department of Biochemistry and Molecular Biology, Faculty of Biology, University of Bucharest, 91–95 Splaiul Independentei, 050095 Bucharest, Romania; bianca.galateanu@bio.unibuc.ro (B.G.); arianahudita@yahoo.com (A.H.)

**Keywords:** biopolymeric spheres, bioproduced antibiotics, antibiotic efficiency

## Abstract

Natural-derived biopolymers are suitable candidates for developing specific and selective performance-enhanced antimicrobial formulations. Composite polymeric particles based on poly(3-hydroxybutyrate-co-3-hydroxyvalerate) and chitosan, P(3HB-3HV)-CS, are herein proposed as biocompatible and biodegradable delivery systems for bioproduced antibiotics: bacitracin (Bac), neomycin (Neo) and kanamycin (Kan). The stimuli-responsive spheres proved efficient platforms for boosting the antibiotic efficiency and antibacterial susceptibility, as evidenced against Gram-positive and Gram-negative strains. Absent or reduced proinflammatory effects were evidenced on macrophages in the case of Bac-/Neo- and Kan-loaded spheres, respectively. Moreover, these systems showed superior ability to sustain and promote the proliferation of dermal fibroblasts, as well as to preserve their ultrastructure (membrane and cytoskeleton integrity) and to exhibit anti-oxidant activity. The antibiotic-loaded P(3HB-3HV)-CS spheres proved efficient alternatives for antibacterial strategies.

## 1. Introduction

Polymers represent essential elements for new and effective nanomedicine platforms, including antimicrobial systems [1,2], drug delivery formulations [3,4], sensing and imaging tools [5,6], architectures for tissue engineering and regenerative medicine [7,8]. The use of polymers in pharmaceutical science is of great interest, starting from their use as excipients in conventional formulations and their ongoing use in modern pharmaceutical formulations.

Polymeric particles showed tremendous results regarding modern drug formulations. Such particular use relies on the intrinsic polymers’ characteristics, such as: (i) bioavailability; (ii) tunable physicochemical, mechanical and thermal behavior; (iii) functionalization and surface (bio)chemical modification; (iv) adjustable solubility and dissolution; (v) circumstantial triggered biodegradability and non-toxic metabolites; (vi) biocompatibility, non-toxicity and non-immunogenicity; (vii) selective ability for specific biological structures; (viii) intrinsic biological activity (antioxidant, anti-inflammatory, antimicrobial, anti-tumor, analgesic, hemostatic, etc.) [9,10,11].

The use of polymers for antimicrobial formulations is of great importance for the healthcare system, due to ineffective conventional antibiotherapy (caused by non-specific and partially selective action) and alarming occurrence of drug-resistant pathogens [12,13]. Antibiotic resistance poses a major threat to global health, and systematic studies to understand the underlying resistance mechanisms and develop performance-enhanced anti-infective formulations are urgently needed.

In this respect, the modification of polymeric particles with synthetic or natural antimicrobial substances provides promising results towards new and effective anti-infective formulations [14,15]. By properly engineering such drug-loaded platforms, polymeric particles (i) act as protective agents for embedded antimicrobials; (ii) facilitate controlled tissue distribution; (iii) exert specific receptor/cell targeting; (iv) determine reduced or mitigated collateral or side effects; (v) provide triggered drug release; (vi) enable local therapeutic effects; (vii) exhibit antimicrobial action at least comparable with the systemic administration of high drug doses [16,17].

Poly(3-hydroxybutyrate-*co*-3-hydroxyvalerate), P(3HB-3HV) or PHBV, is one representative copolymer of natural-derived polyesters with broad applicability in the biomedical field. Besides enhanced mechanical behavior (mechanical shock resistance and flexural strength), thermoplasticity and piezoelectricity, this bacteria-produced copolymer has excellent biocompatibility and biodegradability properties [18,19]. Moreover, the reduced degradation rate of P(3HB-3HV), when compared to other biopolymers, is beneficial for the development of new biomaterials with therapeutic effects, including platforms for tissue engineering (particles, fibers, meshes, films and scaffolds) [20,21,22,23] and smart vehicles for drug delivery [24,25,26,27]. Some intrinsic characteristics of P(3HB-3HV), such as stiffness/flexibility and tensile strength, melting point and glass transition temperature, depend on the ratio between constituent monomers [16,17]. Other features, like porosity, wettability, water and oxygen permeability and biological effects, can be improved though the development of P(3HB-3HV)-based composites [28,29,30].

Chitosan (CS), the second most abundant natural polysaccharide, is a biocompatible, non-toxic, non-immunogenic and biodegradable material, with specific biological properties (including mucoadhesive, antimicrobial, hemostatic and wound healing effects) [31,32]. In addition, the impressive film-forming ability and processability of CS enable its use as an excipient in traditional pharmaceutical tablets, but also for the fabrication of unconventional therapeutic formulations, including micro-/nano-particles, micro-/nano-capsules, fibers and fibrous meshes, films and membranes, (hydro)gels and scaffolds [33,34,35]. Besides enhanced physicochemical features, CS-based composites exert additional properties (enhanced solubility under acidic or alkaline environments [36,37,38], selective receptor coupling or detection [39,40,41]), which are of great interest for advanced biomedical applications.

Bacitracin (Bac), produced by *Bacillus subtilis* and *Bacillus licheniformis* [42], is a polypeptide antibiotic which interferes with the synthesis of cell wall and peptidoglycan in Gram-positive cocci and bacilli [43,44]. Bac dissolves at room temperature in aqueous solution and is highly allergenic and nephrotoxic, therefore it is usually combined with zinc ions to form stable salts or with other antibiotics to enhance its antimicrobial activity [45,46]. Neomycin (Neo) belongs to the family of 2-deoxystreptamine-containing aminoglycoside antibiotics and is an excellent bactericidal agent against Gram-negative bacteria and partially effective against Gram-positive strains [47,48]. Neo, bioproduced by *Streptomyces fradiae*, is also highly allergenic and ototoxic [49] and it was reported to show antiviral activity [50]. Kanamycin (Kan) is produced by *Streptomyces kanamyceticus* and can be used as a mid-term treatment for severe infections and tuberculosis [51,52]. In comparison with previously mentioned antibiotics, the administration of Kan is more varied, but it also possesses certain nephrotoxicity and ototoxicity [53].

Given the attractive characteristics of the above-mentioned natural-derived biopolymers, the aim of the present study was to obtain composite spheres of P(3HB-3HV) and CS to be used as topical biocompatible and biodegradable delivery platforms for the selected bioproduced antibiotics (Bac, Neo and Kan). P(3HB-3HV)-CS spheres showed potentiating effects on the selected bioproduced antibiotics, while they exhibited cytocompatible effects on human-derived fibroblasts (evidenced by quantitative and qualitative assays). Reduced pro-inflammatory effects were evidenced on macrophages treated with antibiotic-loaded composite spheres, evidencing their potential beneficial implications in the wound healing process.

## 2. Materials and Methods

### 2.1. Materials

All reagents used for the synthesis of antibiotic-loaded composite spheres were purchased from Sigma-Aldrich (Merck Group, Darmstadt, Germany), namely poly(3-hydroxybutyric acid-co-3-hydroxyvaleric acid)—P(3HB-3HV) with 8 mol% polyhydroxyvalerate content, high purity chitosan (CS) with ≤40 mol% acetylation degree, polyvinyl alcohol (PVA) with 87–89% hydrolysis degree, bacitracin (Bac) produced by *Bacillus licheniformis* with ≥60,000 U/g (potency), neomycin sulfate (Neo), kanamycin sulfate (Kan) and chloroform.

### 2.2. Synthesis of Antibiotic-Loaded Composite Spheres

Antibiotic-loaded P(3HB-3HV)-CS systems were prepared using a solvent evaporation method [54,55]. Thus, 200 mg P(3HB-3HV) was solubilized in 3 mL CHCl_3_ by sonication. The organic phase was emulsified with a sonicator for 7 min (ON/OFF steps of 5 s and 3 s, limitation temperature of 37 °C) in 15 mL aqueous phase containing 2% (*w/v*) PVA, 1% antibiotics (Bac/Neo/Kan) and CS 1%. After sonication, the emulsion was added in 200 mL deionized water and stirred for 4 h until the complete evaporation of residual CHCl_3_ and then centrifuged at 6000 rpm for 20 min. The obtained spheres were washed four times with ultrapure water, collected by filtration, and finally subjected to freeze drying. Depending on the antibiotic, the resulted systems were denoted as P(3HB-3HV)-CS-Bac, P(3HB-3HV)-CS-Neo and P(3HB-3HV)-CS-Kan.

### 2.3. Characterization Methods

FT-IR spectra were recorded on a Nicolet iN10 MX FT-IR microscope (Thermo Fischer Scientific, Waltham, MA, USA) with an MCT liquid nitrogen cooled detector, in the measurement range 4000–600 cm^−1^. Spectral collection was made in reflection mode at 4 cm^−1^ resolution. For each spectrum, 32 scans were co-added and converted to absorbance using Ominc Picta software (Thermo Scientific).

SEM analysis was performed with a Quanta Inspect F FEI electron microscope (Thermo Fischer Scientific), using secondary electron beams with energies of 30 keV, on samples coated with a thin gold layer.

### 2.4. Microbiological Evaluation of Antibiotic-Loaded P(3HB-3HV)-CS Spheres

To qualitatively assess the antimicrobial potential of the obtained antibiotic-loaded spheres, an adapted diffusion method in nutritive agar was performed. We used 4 bacterial strain models, belonging to Gram-positive (*Staphylococcus aureus* ATCC^®^ 23235 and *Enterococcus faecalis* ATCC^®^ 29212) and Gram-negative (*Escherichia coli* ATCC^®^ 25922 and *Pseudomonas aeruginosa* ATCC^®^ 27853) groups. Briefly, 0.5 McFarland (1–3 × 10^8^ CFU (colony forming units)/mL) suspensions in sterile saline were obtained from overnight cultures, previously cultivated in nutritive agar. The obtained microbial suspensions were utilized to swab inoculate Mueller Hinton agar Petri dishes, as for the disc diffusion technique described in CLSI 2020 standard (https://clsi.org/, accessed on 10 January 2021). Then, 10 µL of each of the obtained microsphere suspensions and controls were drop-added in the inoculated Petri dishes. Antibiotic controls were added at the concentration found in discs, routinely used for disc-diffusion antibiotic susceptibility assay described in the CLSI procedure (30 µg/mL for Kanamycin [56] and Neomycin [57]). Plates were incubated for 20 h at 37 °C and the diameter of growth inhibition was measured.

### 2.5. Biological Evaluation of Antibiotic-Loaded P(3HB-3HV)-CS Spheres

In order to investigate the biocompatibility of simple and antibiotic-loaded P(3HB-3HV)-CS spheres, the human dermal fibroblasts CCD-1070Sk cell line (ATCC^®^ CRL-2091™) was employed. Briefly, cells were cultured in Dulbecco’s Modified Eagle’s Medium (DMEM, Sigma/Merck, Steinheim, Germany), supplemented with 10% fetal bovine serum (FBS, Gibco) and 1% penicillin-streptomycin mixture (Sigma/Merck) all throughout the experiment in standard cell culture conditions (5% CO_2_, 37 °C). One day prior to spheres treatment, CCD-1070Sk cells were seeded in 12-well or 96-well plates at an initial density of 1 × 10^5^ and 0.1 × 10^5^ cells/well, respectively, and incubated overnight to allow cellular attachment. The next day, the culture medium was discarded and replaced with fresh medium for experimental controls and with simple and antibiotic-loaded spheres at a final concentration of 1 mg/mL. Biocompatibility assays were performed as described below after 24 h and 7 days of treatment.

#### 2.5.1. MTT Assay

MTT (3-[4,5-dimethylthiazol-2-yl]-2,5 diphenyl tetrazolium bromide) assay (Sigma/Merck) was employed to evaluate the cellular metabolic activity of CCD-1070Sk cells after spheres treatment as an indicator of cellular viability. Briefly, the culture medium and treatments were discarded, replaced with a freshly prepared solution of MTT (1 mg/mL) and incubated for 4 h in the dark in standard conditions. The obtained formazan crystals were subsequently dissolved in isopropanol (Sigma/Merck) and the optical density of the resulted solution was measured at 550 nm using the FlexStation III multimodal reader (Molecular Devices, San Jose, CA, USA).

#### 2.5.2. LDH Assay

LDH assay was performed in order to evaluate the potential cytotoxicity of the spheres on CCD-1070Sk cells. In this view, media samples were collected from control and microsphere treated CCD-1070Sk monolayers and mixed with the components of the “*in vitro* toxicology assay kit lactate dehydrogenase based TOX—7” kit (Sigma/Merck) according to the manufacturer’s recommendations. The resulted solution was measured at 490 nm using the FlexStation III multimodal reader (Molecular Devices, San Jose, CA, USA).

#### 2.5.3. Cell Morphology Evaluation

To investigate the global morphology of human dermal fibroblasts exposed to spheres treatment, the F-actin filaments were stained with phalloidin. Briefly, the cellular monolayers were washed with PBS (Sigma/Merck) and fixed for 15 min using a 4% paraformaldehyde solution (Sigma/Merck). After cell membrane permeabilization with a 0.1% Triton X-100 solution in 2% BSA (Sigma/Merck), the CCD-1070Sk monolayers were incubated in the dark for 1 h at 37 °C with a phalloidin-FITC solution (Sigma/Merck). Before microscopy visualization, cells were counterstained for 10 min with DAPI to highlight cell nuclei. Fluorescence was investigated using Olympus IX73 inverted microscope with fluorescence modulus (Olympus, Tokyo, Japan) and images were captured and processed using CellSense F software.

#### 2.5.4. ROS Production Investigation

The impact of the spheres treatment on reactive oxygen species (ROS) production was measured using ROS-Glo H_2_O_2_ assay (Promega, Madison, WI, USA). Briefly, for the final 6 h of treatment for both experimental time points, the H_2_O_2_ substrate was added at a final concentration of 25 μM and the cell cultures were further incubated at 37 °C in a humidified atmosphere of 5% CO_2_. Afterwards, 100 μL of ROS-Glo Detection Solution was added and the plate was incubated 20 min at room temperature. The luminescence of final solutions was measured using the Flex Station III multimodal reader (Molecular Devices).

All spectrophotometric and luminescence data were statistically analyzed using GraphPad Prism software (San Diego, CA, USA) and results are represented as a mean ± S.D. of 3 independent experiments. The statistical significance (* *p* ≤ 0.05) was determined using the non-parametric two-way ANOVA algorithm, Bonferroni test.

#### 2.5.5. Proinflammatory Potential Assessment

The proinflammatory potential of the spheres was investigated using the RAW 264.7 macrophage cell line (ATCC), maintained in culture in DMEM, supplemented with 10% FBS and 1% antibiotic mixture. RAW 264.7 cells were seeded in 96-well plates at an initial density of 2.5 × 10^4^ cells/cm^2^ and incubated for 24 h. After confirming cellular attachment, the RAW 264.7 monolayers were serum-deprived and stimulated with lipopolysaccharide from *Escherichia coli* O111:B4 (LPS, 10 μg/mL, Sigma/Merck) for 1 day before spheres treatment, to simulate an existing inflammatory process. After 6 and 24 h of treatment with simple and antibiotic-loaded spheres, media samples were collected from all experimental conditions and further investigated for measuring the nitric oxide (NO) production and various inflammation-related cytokine levels. The NO production was determined using the Griess reagent system assay (Promega) based on the Griess reaction [58], according to the manufacturer’s instructions. Firstly, 50 μL of the collected cell culture supernatants were mixed with 50 μL Sulfanilamide solution and incubated at room temperature in the darkness for 20 min. At half the incubation time, 50 μL of *N*-1-naphthyl ethylenediamine dihydrochloride (NED) solution was added. In the end, the optical density of the resulting solution was read at 550 nm using Flex Station III microplate reader (Molecular Devices). The nitrite concentration was extrapolated from a nitrite standard reference curve that was prepared according to the instruction available in the kit’s datasheet. The obtained data were further statistically analyzed using GraphPad Prism software and results are represented as a mean ± S.D. of 3 independent experiments. The statistical significance (* *p* ≤ 0.05) was determined using the non-parametric two–way ANOVA algorithm, Bonferroni test.

Furthermore, the collected media samples were investigated by flow cytometry to quantify various cytokine levels using a bead based multiplex assay (BD CBA Inflammation Kit, Becton Dickinson, NJ, USA). The following cytokine protein levels were investigated: Interleukin-6 (IL-6), Interleukin-10 (IL-10), Interferon-γ (IFN-γ), Tumor Necrosis Factor (TNF—α), and Interleukin-12p70 (IL-12p70). In this view, the harvested media samples were processed according to the manufacture’s indications. Briefly, 50 μL of sample was incubated for 2 h at room temperature and darkness with 50 μL of IL-6, IL-10, IFN-γ, TNF-α and IL-12p70 mixed Capture Beads and 50 μL Inflammation PE Detection Reagent. After a wash step, all tubes were analyzed in a Cytoflex (Beckman Coulter, Brea, CA, USA) flow cytometer using CytExpert Data for sample acquisition and data analysis. The graphical representation of the obtained results was performed using the GraphPad Prism software. The mean of the obtained data was obtained from three independent experiments and is presented as the arithmetic mean ± S.D. The statistical significance (* *p* ≤ 0.05) was determined using two–way ANOVA algorithm for group comparison, Bonferroni test.

## 3. Results and Discussions

### 3.1. Physicochemical Characterization

Emulsification/solvent evaporation represents an efficient, facile and versatile method to obtain biopolymer systems (particles, spheres, capsules) for unconventional pharmaceutical formulations [59,60]. This method is suitable to develop micro-/nano-carriers for various compounds, including inorganic nanostructures [61,62], antimicrobial agents [63], chemodrugs [64,65] and liposoluble drugs [66,67].

**Figure 1:**
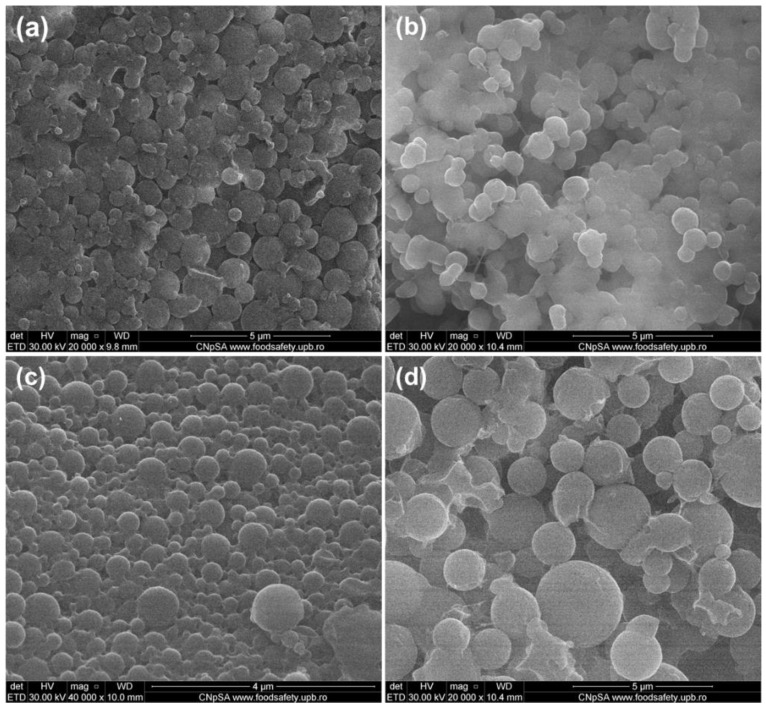
SEM micrographs of (**a**) P(3HB-3HV)-CS, (**b**) P(3HB-3HV)-CS-Bac, (**c**) P(3HB-3HV)-CS-Neo and (**d**) P(3HB-3HV)-CS-Kan spheres.

The morphology of pristine and antibiotic-loaded P(3HB-3HV)-CS samples was investigated by SEM (Figure 1). Regardless of the composition, aggregates of spherical particles were obtained. Similar dimensions (diameters around 600 nm) were noticed in the case of simple and Bac-loaded P(3HB-3HV)-CS. Particle sizes around 400 nm and 1 µm were evidenced for P(3HB-3HV)-CS-Neo and P(3HB-3HV)-CS-Kan systems, respectively. The obtaining of nano-/micro-aggregates was previously reported for CS [68,69] and polyester/CS [70,71], as higher concentrations of CS are related with increased hydrogen bonds between CS molecules and electrostatic interactions with organic salts.

**Figure 2:**
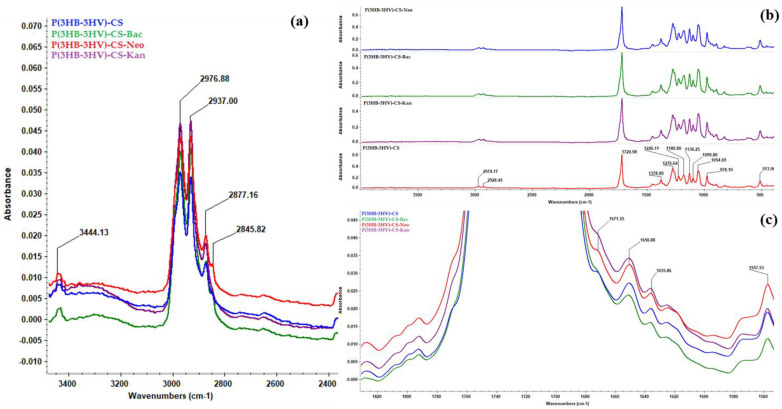
General IR spectra of P(3HB-3HV)-CS, P(3HB-3HV)-CS-Bac, P(3HB-3HV)-CS-Neo and P(3HB-3HV)-CS-Kan spheres (**b**), and zoomed in regions to evidence C-H (**a**) and amide (**c**) vibrations.

Individual and overlapped IR spectra (Figure 2a–c, respectively) were recorded from different points for all synthesized spheres. The presence of P(3HB-3HV) was confirmed through the following absorption bands: ~1720 cm^−1^ (strong stretching vibrations of C=O ester group, particularly associated with the crystalline phase of copolymer), ~1378 cm^−1^ (symmetric expansion of CH_3_ groups), ~1276 cm^−1^ (C–H stretching), ~1054 cm^−1^ (C–O–C stretching vibrations) and ~978 cm^−1^ (C–C stretching) [72,73], all evidenced in Figure 2b. Being the main constituent of proposed spheres, the IR maxima identified for P(3HB-3HV) covered some of the IR bands of CS, such as ~1378 cm^−1^ (overlapped C–N stretching and C–H asymmetric bending) and ~1054 cm^−1^ (C–O–C stretching vibrations). The zoomed-in IR spectra from Figure 2c evidences the presence of specific CS maxima at ~1671 cm^−1^ (amide II), ~1650 cm^−1^ and ~1635 cm^−1^ (amide I doublet) and ~1557 cm^−1^ (C–O skeletal vibrations) [74,75]. Moreover, the IR doublets from ~2976 cm^−1^/~2937 cm^−1^ and ~2877 cm^−1^/~2845 cm^−1^ wave numbers (Figure 2a) correspond to the C–H stretching vibrations from –CH_3_ and –CH_2_– moieties of P(3HB-3HV) and CS, while the ~3444 cm^−1^ maxima was assigned to the overlapped stretching of –NH_2_ and –OH functions from CS [76,77]. The absorption band at 514 cm^−1^ confirmed the presence of PVA.

### 3.2. Antimicrobial Activity

The antimicrobial character of obtained spheres was analyzed using four types of reference strains, of which there were two Gram-negative strains (*E. coli*, *Ps. aeruginosa*) and two Gram-positive strains (*S. aureus*, *E. faecalis*). All considered bacterial species have a great biomedical impact, being involved in community and nosocomial infections [78,79] while developing increased antibiotic resistance rates in the last decades [80,81].

**Figure 3:**
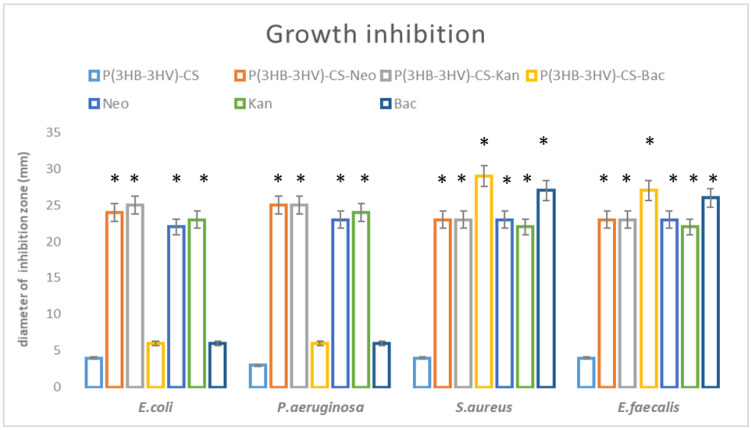
Graphic representation of antimicrobial potential of the obtained spheres, represented as diameter of inhibition zone evaluated for *E. coli*, *E. faecalis*, *P. aeruginosa* and *S. aureus* after 20 h incubation. * *p* < 0.05 (control sample = P(3HB-3HV)-CS was compared to antibiotic containing samples).

Growth inhibition results showed that the obtained spheres could enhance the effects of antibiotics against bacteria, as presented in Figure 3. The diameters of growth inhibition were higher in the case of P(3HB-3HV)-CS-Neo and P(3HB-3HV-CS-Kan spheres, as compared to the plain antibiotic controls (Kan and Neo). The most enhanced antimicrobial effect was observed for the Gram-negative species, *E. coli* and *P. aeruginosa*, as the diameter of growth inhibition was significantly increased for both antibiotics. On the other hand, the ability of obtained spheres to enhance the antibiotic potential against Gram-positive strains is lower. We have obtained increased values of the diameter of inhibition zones only for Kan-embedded polymeric spheres, as compared with the control (plain kanamycin solution). P(3HB-3HV)-CS-Bac spheres proved their potentiating effects on the bacitracin action against Gram-positive bacteria. The ability of the proposed composite system to exhibit enhanced antibacterial activity was increased for both *S. aureus* and *E. faecalis* tested strains.

Even if the antibiotics alone inhibit bacteria, any change in the diameter zone of antibiotics and antibiotic loaded-systems should be reported and further investigated, since it is well known that even 1–2 mm difference in the diameter of growth inhibition could make the difference among Resistant and Susceptible pattern (https://clsi.org/, accessed on 10 January 2021). In our future studies we will apply other qualitative methods, such as MIC (minimum inhibitory concentration assay), so we can find out the precise decrease in the antibiotic concentration, which is necessary to reveal an antimicrobial effect for each bacteria species.

### 3.3. Biological Activity

Despite the emerging need for developing efficient anti-infective therapeutic strategies, the biocompatibility of newly designed antibiotic delivery platforms is a mandatory step that needs to be addressed for their prospective use. As human dermal fibroblasts represent key players [82,83] in the wound healing process, the CCD—1070Sk cells were selected as an *in vitro* cellular model for assessing the cytotoxic potential of herein proposed antibiotic- loaded spheres. Therefore, CCD—1070Sk cells were exposed for 24 h and 7 days to simple and Bac/Neo/Kan- loaded P(3HB-3HV)-CS spheres.

**Figure 4:**
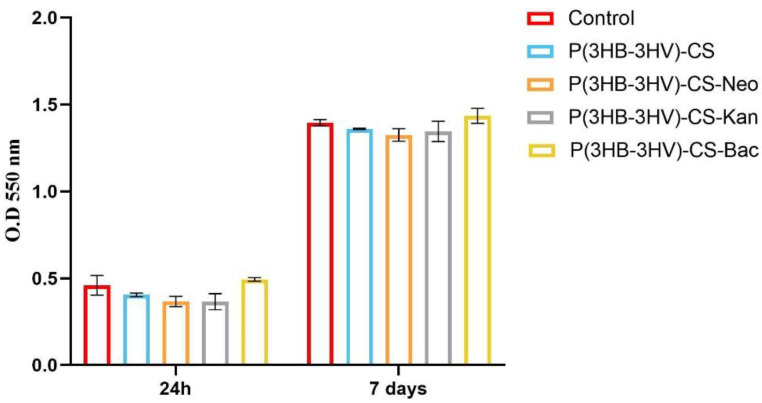
Graphic representation of human dermal fibroblasts viability after 24 h and 7 days of exposure to simple and Bac-/Neo-/Kan-loaded P(3HB-3HV)-CS spheres as revealed by the MTT assay. The represented data are the mean values of three independent experiments ± S.D.

The CCD—1070SK cellular metabolic activity after microsphere treatment was assessed by the MTT assay, as an indicator of cell viability and proliferation (Figure 4). In comparison with controls, human dermal fibroblasts exposed to P(3HB-3HV)-CS spheres treatment presented no significant differences of the cellular viability at the investigated time points. The addition of selected antibiotics within the spheres’ structure did not impact the viability of CCD—1070Sk cells, as no notable changes of cellular viability were observed as compared to the untreated control after 24 h and 7 days of exposure. Moreover, the examined spheres sustained human dermal fibroblasts proliferation, as highlighted by the statistically significant increase (*p* ≤ 0.0001) of the cellular viability after 7 days of exposure to treatment as compared with the 24 h viability for all screened samples.

**Figure 5:**
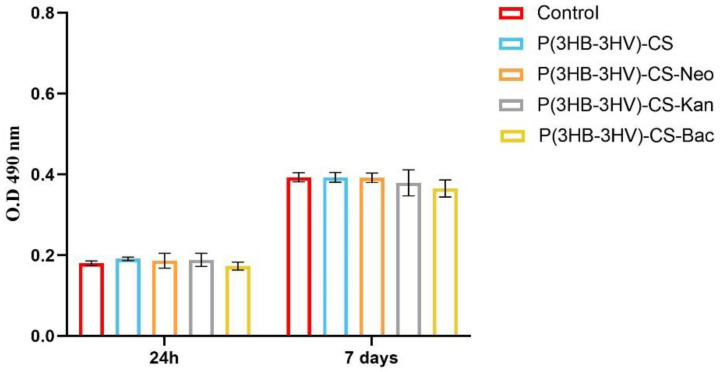
Graphic representation of the LDH levels released in the culture medium by human dermal fibroblasts after 24 h and 7 days of treatment with simple and Bac-/Neo-/Kan-loaded P(3HB-3HV)-CS spheres. The represented data are the mean values of three independent experiments ± S.D.

Cell membrane integrity after spheres treatment was investigated by quantifying the LDH released in the culture medium as response to the CCD-1070Sk cells exposure to bare and antibiotic-loaded P(3HB-3HV)-CS spheres. After 24 h, no significant variations of LDH levels were detected in the CCD-1070Sk cultures grown in presence of simple and Bac-/Neo-/Kan-loaded spheres, as compared to the control cells (Figure 5). A similar pattern of the released LDH levels was observed after 7 days of culture, highlighting that the designed antibiotic delivery platforms lack the ability to alter the structural integrity of the cellular membrane.

**Figure 6:**
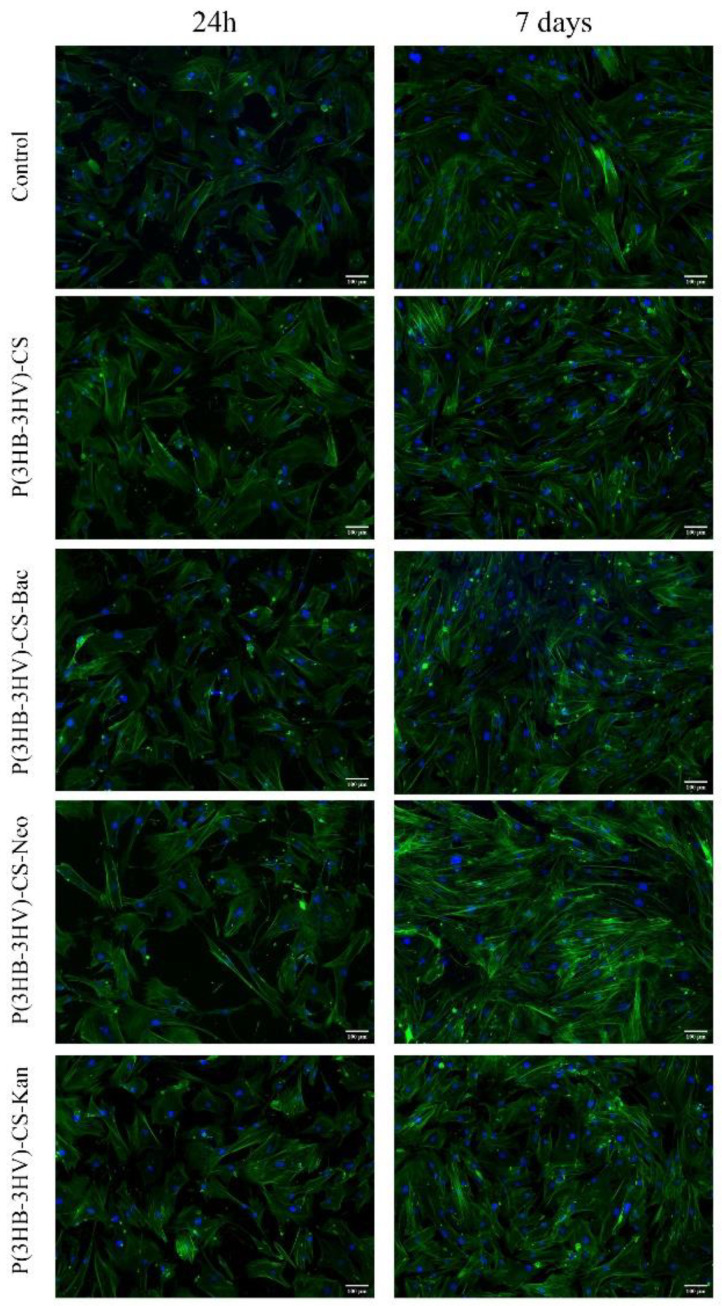
Fluorescence micrographs revealing the cellular morphology of the human dermal fibroblast treated for 24 h and 7 days with simple and Bac-/Neo-/Kan-loaded P(3HB-3HV)-CS spheres. F-actin filaments are stained with phalloidin-FITC (green) and nuclei are stained with DAPI (blue). Scale bar = 100 µm.

In order to investigate the potential of obtained spheres to alter the normal cell structure of human dermal fibroblasts, the cytoskeleton of CCD-1070Sk cells was investigated post-treatment by fluorescence microscopy after phalloidin-FITC and DAPI staining. No differences between the assembly of actin filaments of untreated human dermal fibroblasts cell cultures and treated cells were noticed, independent of the applied treatment (Figure 6). After 24 h, CCD-1070Sk cells cultured in the presence of simple and antibiotic-loaded spheres presented a spindle-like morphology identical to the untreated cells. Moreover, after 7 days of treatment, the human dermal fibroblasts uniformly spread on the culture dish under all investigated experimental conditions, while maintaining their typical spindle-like morphology characterized by long and well-defined actin filaments and the ability to form strong intercellular compact networks.

**Figure 7:**
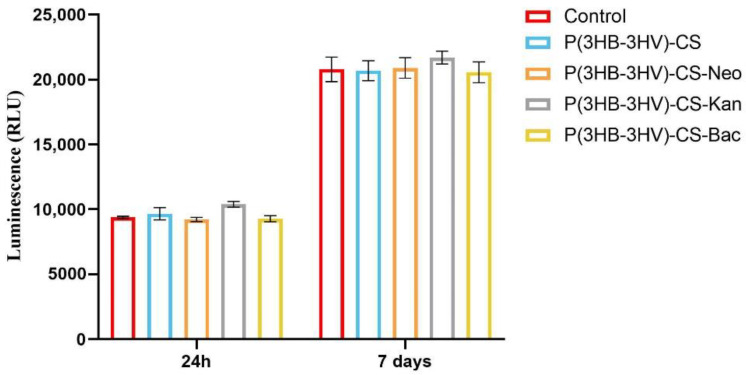
Graphic representation of the H_2_O_2_ levels in human dermal fibroblasts cell cultures exposed for 24 h and 7 days at simple and Bac-/Neo-/Kan-loaded P(3HB-3HV)-CS spheres treatment. The represented data are the mean values of three independent experiments ± S.D.

The potential of pristine and antibiotic-loaded P(3HB-3HV)-CS spheres to trigger ROS production in human dermal fibroblast cell cultures was investigated by measuring H_2_O_2_ production as an indicator of oxidative stress, using a ROS-Glo H_2_O_2_ Assay. The basal level of H_2_O_2_ detected in the untreated CCD-1070Sk cultures was similar to the H_2_O_2_ levels detected in the human fibroblast cultures exposed to simple and Bac-/Neo-/Kan-loaded spheres treatment at both experimental time points, showing that the spheres treatment did not induce oxidative stress (Figure 7). In general, healthy cells exposed to bacterial antibiotics show enhanced ROS production, aminoglycosides being responsible for causing mitochondrial dysfunction. In time, the overproduction of ROS causes tissue oxidative damage, one of the underlying causes in developing adverse effects as a response to prolonged use of antibiotics [84,85]. The obtained results showed that the use of P(HB-3HV)-CS spheres as delivery platforms for bioproduced antibiotics do not trigger H_2_O_2_ overproduction, highlighting that the novel Bac-/Neo-/Kan-loaded microspheres do not induce oxidative stress in the CCD-1070Sk cell cultures.

**Figure 8:**
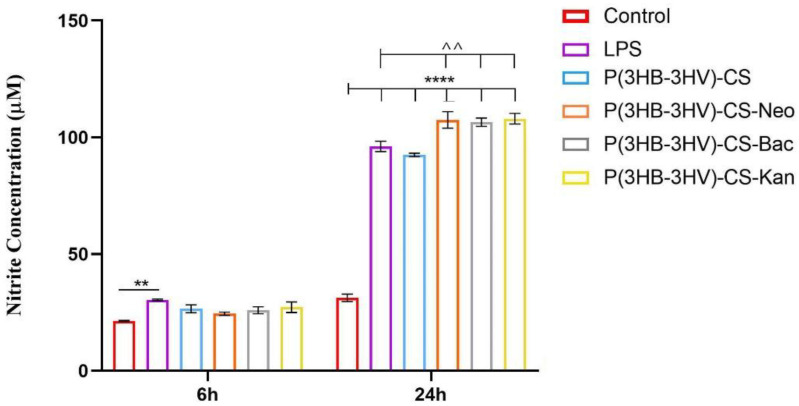
Graphic representation of the nitrite levels as a measure of NO release in LPS-activated RAW 264.7 macrophages cell cultures exposed for 6 h and 24 h to simple and Bac-/Neo-/Kan-loaded P(3HB-3HV)-CS spheres treatment. The represented data are the mean values of three independent experiments ± S.D. (** *p* ≤ 0.01 LPS vs. control; ^^ *p* ≤ 0.01 sample vs. LPS; **** *p* ≤ 0.0001 sample vs. untreated control).

The macrophage RAW 264.7 cell line is a well-studied model for *in vitro* inflammation studies as these cells can easily be stimulated with LPS to mimic inflammatory conditions [86]. In this view, LPS from *E. coli* was used to stimulated RAW 264.7 cells to produce NO as macrophages release high levels of NO in response to exposure to bacterial products [87]. Therefore, the Griess reagent was used to measure the NO productions in LPS-activated RAW 264.7 cell cultures exposed to simple and Bac-/Neo-/Kan-loaded P(3HB-3HV)-CS spheres treatment (Figure 8). After 6 h of treatment, no significant differences in the NO release were observed in cell cultures exposed to simple or antibiotics-loaded microspheres and control cell cultures. In contrast, after 24 h a statistically significant increase of the NO production (*p* ≤ 0.0001) was observed in all LPS-activated macrophage cultures compared with the unstimulated control cells, independent of the applied treatment. Furthermore, the Bac-/Neo-/Kan-loaded P(3HB-3HV)-CS spheres treatment increased the NO production as the nitrite concentration was significantly higher (*p* ≤ 0.01) under these experimental conditions as compared with simple or pristine P(3HB-3HV)-CS sphere-treated LPS activated macrophages. This capacity of the antibiotics loaded P(3HB-3HV)-CS spheres to augment the NO production could be beneficial since the NO is a key mediator of the wound healing process and of antimicrobial mechanisms that decrease bacterial loads at wound sites [88].

**Figure 9:**
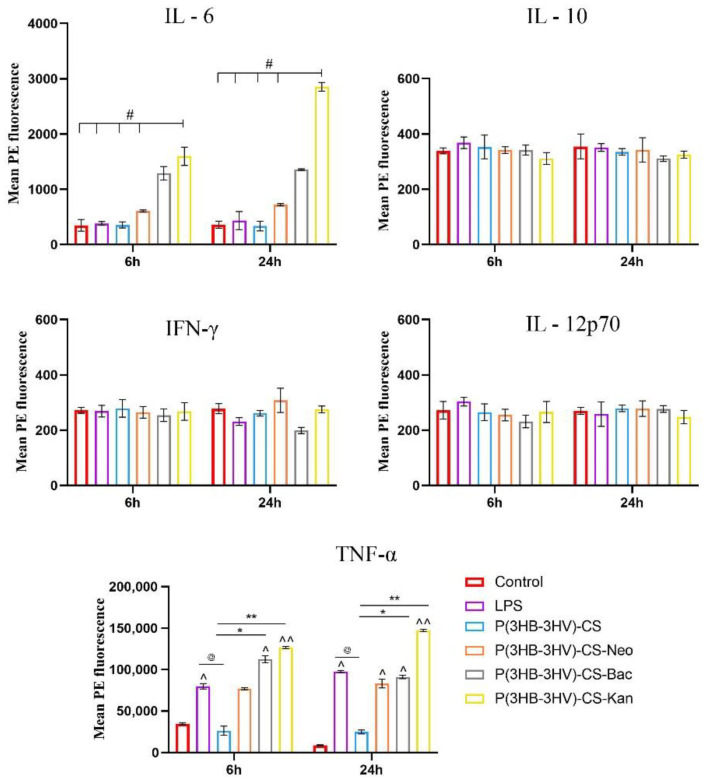
Graphic representation of IL-6, IL-10, IFN-γ, IL-12p70 and TNF-α cytokine levels released by LPS-activated RAW 264.7 macrophages after 6 h and 24 h of exposure to simple and Bac-/Neo-/Kan- loaded P(3HB-3HV)-CS spheres treatment. The experimental control was represented by media samples collected from non-stimulated RAW 264.7 cell cultures (# *p* ≤ 0.05 P(3HB-3HV)-CS-Kan vs. control and samples; ^ *p* ≤ 0.05 and ^^ *p* ≤ 0.01 sample vs. control; @ *p* ≤ 0.05 samples vs. LPS; * *p* ≤ 0.05 and ** *p* ≤ 0.01 samples vs. P(3HB-3HV)-CS). The represented data are the mean values of three independent experiments ± S.D.

In infections, activated macrophages trigger various microbicidal mechanisms by releasing proinflammatory cytokines, such as TNF-α, IL-6 or IL-1, that fight against the existing pathogens in order to restore tissue homeostasis [89]. Moreover, activated macrophages are involved in the inflammatory and proliferative stages of the wound healing process [90]. Therefore, LPS-activated RAW 264.7 macrophages were used for investigating the potential of obtained spheres to augment cytokine production. Both LPS activation and exposure to simple and antibiotic-loaded spheres did not stimulate the production of IL-10, IFN-γ and IL-12p70 in RAW 264.7 cell cultures, as no significant differences between samples were identified (Figure 9). Regarding the expression of IL-6 and TNF-α proinflammatory cytokines, the spheres treatment affected the LPS-activated cytokine profile in an antibiotic type-dependent manner. The expression of IL-6 was significantly increased (*p* ≤ 0.05) and only in LPS-activated macrophages were treated with P(3HB-3HV)-CS-Kan spheres. IL-6 secretion is essential for a proper wound healing process as it triggers additional cytokine release, downstream events that stimulate the migration of fibroblasts to the injury sites and sustain the transition of activated macrophages to M2 phenotype [91]. TNF-α is a proinflammatory cytokine that is quickly released by macrophages as response to LPS stimulation, as highlighted by the significant increased levels (*p* ≤ 0.05) observed in LPS-RAW 264.7 culture cells in comparison with the control culture cells after 6 h. The treatment with antibiotic-loaded spheres stimulated TNF-α overproduction starting with 6 h of treatment, as a 1.4-fold and 1.6-fold increase of TNF-α expression was identified in RAW 264.7 activated cells exposed to P(3HB-3HV)-CS-Neo and P(3HB-3HV)-CS-Kan spheres in comparison to LPS-activated cells cultures. After 24 h of treatment, only the Kan-loaded spheres maintained their potential to stimulate TNF-α secretion more than LPS stimulation. Interestingly, the unloaded spheres severely reduced the TNF-α levels triggered by the LPS-activation (*p* ≤ 0.05), an aspect that can be attributed to the anti-inflammatory potential of the chitosan [92]. The obtained spheres are not able to induce inflammation or present low proinflammatory potential in the case of Kan-loaded spheres. The proinflammatory TNF-α cytokine is involved in the early process of wound healing by recruiting inflammatory cells to the site of infection and stimulating macrophage phagocytic activity [93,94]. Therefore, stimulation of TNF-α production by P(3HB-3HV)-CS-Neo/-Kan treatment could contribute to an enhancement of the wound healing process, where increased levels of TNF-α are imperative for proper repair.

Biomaterials based on P(3HB-3HV) and CS proved beneficial for the development of new and performance-enhanced pharmaceutical formulations. For example, highly stable PHBV-PEG (polyethylene glycol) particles proved efficient encapsulation systems for epirubicin and exhibited pH-dependent drug’s release, with fast and sustained release under neutral and acidic conditions, respectively. Enhanced antibacterial effects were reported for proposed composite nanoparticles (152.3 ± 0.6 nm), when compared to equivalent concentrations of free drug [95]. Alginate coatings incorporating levofloxacin-loaded PHBV microspheres (2–3 μm particle size) determined efficient bactericidal effects of metallic materials [96]. PHBV particles were also assessed as efficient encapsulation platforms for anti-oxidant [97,98] and anti-tumor [99,100] agents, which further determined long-term and potentiated therapeutic effects.

Positively charged CS nanoparticles [101] and CS-decorated liposomes [102] were successfully used for the encapsulation and prolonged release of natural-derived cinnamaldehyde, while they exhibited fast bactericidal effects against Gram-positive pathogens. Highly stable albumin corona-modified CS nanosystems loaded with carvacrol showed promising results for the treatment of salmonellosis, due to enhanced encapsulation and delayed release of carvacrol and limited degradation under gastrointestinal simulated conditions [103]. CS nanosystems embedded with antibiotic-functionalized magnetite nanoparticles showed potentiating effects on aminoglycosides [104,105], cephalosporins [106] and quinolones [107], while maintained highly biocompatible behavior for healthy human cells. Moreover, composite particles of CS were evaluated as cytocompatible platforms for the controlled and stimuli-responsive release of commercial antibiotics [108,109,110]. CS, which was classified as generally safe by the US Food and Drug Administration, was successfully evaluated as a biocompatible and effective delivery platform for various therapeutic biosubstances, including anti-oxidants [111,112], antivirals [113,114] and chemodrugs [115,116].

Given the excellent biological-related properties and effects of P(3HB-3HV) and CS, their composites are of great interest for biomedical application, particularly for tissue engineering and regenerative medicine uses. While P(3HB-3HV)-based complex architectures are thoroughly investigated for hard tissue restoration and regeneration [117,118], CS-based biomaterials possess an impressive and extensive potential to be used for both hard [119,120] and soft [121,122] tissue repair and healing.

Electrospun scaffolds of P(3HB-3HV) incorporated with CS and hydroxyapatite showed osteoconductive potential and an apatite-like mineralization ability [123], while composite scaffolds based on P(3HB-3HV), CS and calcium sulfate hemihydrate exhibited osteogenic activity on bone marrow stromal cells and promoted new bone formation [124]. Chondroitin sulfate nanoparticles loaded within CS-P(3HB-3HV) composite hydrogel induced chondrogenic differentiation of mesenchymal stem cells, thus exhibiting great potential for nucleus pulposus tissue engineering [125]. Moreover, P(3HB-3HV)/CS composite mats [126] and membranes [127] were successfully assessed for wound healing applications.

The herein proposed P(3HB-3HV)-CS spheres showed the potentiating effects on bioproduced antibiotics: bacitracin (Bac), neomycin (Neo) and kanamycin (Kan), while sustaining the proliferation and normal growth of human-derived fibroblast cells. Moreover, the composite spheres did not affect the normal cellular ultrastructure and did not induce oxidative stress in fibroblasts cultures. The non-toxic effects of antibiotic-loaded biodegradable polyester particles on fibroblasts, while preserving or enhancing their antibacterial activity, was also reported by other studies [128,129,130]. Bac-/Neo-loaded composite spheres did not have a proinflammatory effects on macrophages, while P(3HB-3HV)-CS-Kan exhibited reduced proinflammatory effects, as evidenced by the levels of IL-6 and TNF-α (essential cytokines for the proper wound healing process) [131,132].

## 4. Conclusions

In this study we aimed to design a biocompatible and biodegradable composite system, able to enhance the delivery and efficiency of bioproduced antibiotics. Spheres composed of PHBV and CS proved significant potentiating effects on common antibiotics, being effective against both Gram-positive and Gram-negative species. The antibiotic-loaded P(3HB-3HV)-CS spheres showed absent or reduced proinflammatory effects on macrophages, while exerting no cytotoxic effects on dermal fibroblast and no alterations of the cellular microstructure. These properties, along with their good biocompatibility and natural origin, recommend the obtained composite systems as efficient candidates for alternative antibacterial strategies. Future experiments aiming to investigate their degradability and ecological safety will be performed in order to confirm their impact as ecological antimicrobials.

## Data Availability

The data presented in this study are available on request from the corresponding author.
